# Performance of Forensic Age Estimation by Aspartic Acid Racemization and DNA Methylation: A Systematic Review

**DOI:** 10.12688/f1000research.147348.2

**Published:** 2025-12-16

**Authors:** Eko Prastyo, Elza Ibrahim Auerkari, Antonius Winoto Suhartono, Roben Suhadi Pasaribu, Achmad Gigih Andy Putra, Pertti Auerkari

**Affiliations:** 1 Forensic Odontology Specialist Study Program, Department of Oral Biology, University of Indonesia, Depok, West Java, Indonesia; 2 Division of Forensic Odontology, Oral Biology, University of Indonesia, Depok, West Java, Indonesia; 3 Master's Programme in Dental Sciences, Division of Forensic Odontology, University of Indonesia, Depok, West Java, Indonesia; 4 Master’s Programme in Biomedical Sciences, Faculty of Medicine, University of Indonesia, Depok, West Java, Indonesia; 5 Department of Mechanical Engineering, School of Engineering, Aalto University, Aalto, Finland

**Keywords:** Age estimation, aspartic acid racemization, DNA methylation, forensics

## Abstract

**Background:**

Forensic age estimation is not difficult when the body is found in good condition, but in cases of severe decomposition or damage, such as burnt or separated body parts, then the analysis can only be done with bones and teeth. There has been abundant research and development in the field of related biochemistry over the years. Various molecular changes occur in hard tissues and long-lived proteins, such as those in bones and teeth during the physiological process of aging. Aspartic acid racemization and DNA methylation are still the most frequently used age estimation methods because of their advantages in accuracy.

**Method:**

This study aimed to compare the accuracy of DNA methylation and aspartic acid racemization methods for age estimation. Journal articles were searched in the electronic databases PubMed, Scopus, and Semantic Scholar of 2017-2022 according to PRISMA guidelines.

**Result:**

Twelve journal articles were eligible for review. The DNA methylation method is quite simple to use because of commercially available methylation kits. Furthermore, the results can be obtained relatively quickly without requiring many samples, and the method is less sensitive to thermal and other damage than the aspartic acid racemization method.

**Conclusion:**

The aspartic acid racemization method for age estimation has high accuracy, especially in determining age at death. However, temperature and the condition of the teeth affect the racemization of aspartic acid. Given that DNA methylation is generally stable in a wide range of temperatures, it provides a better approach to determining the chronological age even from charred remains.

## Background

Forensic age estimation is not particularly challenging when the body is found in decent condition. However, when the body is severely decomposed or damaged, such as consisting of separated or burnt body parts, age estimation can only be done by using features and material samples of bones and teeth. ^1, 2^ Several methods are used to estimate age, such as the closure of the cranial sutures, changes in the ends of the sternal bones, and morphological changes in the symphysis pubis and the surface of the auricular. However, the skill and experience of the person analyzing the body determine the accuracy of these traditional methods, and they have a wide range of estimation errors, from 10 to 20 years. ^3^

Developmental methods such as the dimensions of the bones, the presence of ossification centers, the fusion of the epiphyses, the mineralization of the teeth, and the eruption of the teeth can be used to estimate age very accurately in childhood. After childhood, the transitional stage is distinguished by the cessation of bone growth and development (between the ages of 20 and 25). There are also several age-related characteristics, such as the fusion of the sternoclavicular, iliac crest epiphyses, loss of fronto-sphenoid sutures and spheno-occipital or basilar synchondrosis, and the development of third molars. For adults, several morphological methods use teeth, symphysis pubis, fourth rib, auricular surface, and acetabulum to assess age. ^4^

Protocols for age estimation using morphological methods are currently being withdrawn because the results obtained are inaccurate and provide a margin of error of greater than ten years. Therefore, over time biochemical methods have received increasing and wider attention for development. ^5^ Age estimation can be conducted by using several molecular biomarkers, including aspartic acid racemization, DNA damage response, DNA methylation, T-cell DNA rearrangement, mitochondrial DNA mutations, advanced glycation end products (AGEs), and telomere shortening. ^6, 7^ Physiological aging involves molecular changes in long-lived proteins and hard tissues including teeth and bones. Of such methods, aspartic acid racemization and DNA methylation are still most frequently used for age estimation because of their advantages in terms of accuracy. ^3^

## Methods

### Study protocol

This research followed the guidelines of Preferred Reporting Items for Systematic Reviews and Meta-Analyses (PRISMA) ^8^ ( https://doi.org/10.5281/zenodo.13624062).

### Eligibility criteria and exclusion criteria

The inclusion criteria were as follows: (1) journal is an original article, (2) journal was published within the last 5 years, (3) journal can answer question from PICO. The approach of PICO (Population-Interventions-Control-Outcomes) was used to define the eligibility criteria. The following criteria were used: P: people of all age groups and genders; I: aspartic acid racemization method; C: DNA methylation method and O: age estimation.

Exclusion criteria were as follows: (1) Text is not original research (case reports, narrative reviews, controlled clinical trials, or case series), (2) experiments involving experimental animals, (3) texts not available in full, and (4) texts written in a language other than English.

### Search strategy and information sources

Systematic electronic literature searches were performed in Scopus, MEDLINE/PubMed, and Semantic Scholar databases for literature published in 2017-2022. The last electronic search was performed on 28 December 2022. The literature search strategies are shown in Table S 1 (Refer extended data).

### Study selection

E.P. conducted an initial literature evaluation and selection. Subsequently, the articles were evaluated and approved by R.S.P and A.G.A.P. The article selection process consisted of reducing duplicates, after which the articles were screened for the above-mentioned keywords in their titles and abstracts. The second screening was conducted according to the inclusion and exclusion criteria to find relevant full texts, and we selected articles that met the requirements. Then, the full texts were reviewed (read and graded) by all three reviewers to retrieve relevant data. Discussion and consensus were used to resolve disagreements between reviewers.

### Data synthesis

Three reviewers synthesized data independently to avoid bias when conducting reviews. The data synthesized included the name of the first author of the journal, year of publication, source and the number of samples, research location, method, treatment of aspartic acid racemization (preparation and laboratory techniques), and treatment of DNA methylation (DNA extraction technique, bisulfide treatment, and target genes), examination techniques, research results, conclusions, and research limitations.

### Risk of bias

To ensure quality and minimize the risk of bias, the authors conducted a study evaluation based on a scoring tool that assesses the quality of a systematic review study. ^9^ This checklist consists of 11 components and can aid inclusion in Systematic Review studies. ^9^

## Results

### Literature search and description

Searches were performed on three electronic databases: PubMed/MEDLINE (n = 154), Scopus (n = 255), and Semantic Scholar (n = 19) using the BOOLEAN system to find a total of 428 journal articles. Out of 428 articles, 299 were automatically excluded by the system for being published before 2017 and 53 were excluded as they were duplicates, bringing the total number of articles to 76. Automatic filtering was performed to exclude 32 articles that were not research articles, bringing the total to 44 articles. With title and abstract screening to find articles that match the inclusion criteria, 23 articles were excluded, bringing the total number to 21. Twenty-one articles were read in full, and 9 did not meet the criteria or did not answer the PICO. The final number of articles to be reviewed was 12. The flowchart of this study is explained in Figure S1 (Refer extended data) below.

### Data synthesis and results

The results of the literature synthesis include the first author, year of publication, sample source, number of samples, population, method, treatment (DNA extraction technique, bisulfide treatment, preparation stage, and laboratory stage), examination technique, results, conclusions, and research limitations (Table S 2). Refer extended data.

Of the 12 journal articles, research was conducted in several countries such as Japan (n = 2), China (n = 1), Germany (n = 1), Poland (n = 1), Austria (n = 1), Spain (n = 3), Scandinavia (n = 1), Pakistan (n = 1), and Kuwait (n = 1). Eleven articles used teeth as a sample source to be tested and 1 study used blood as a sample source.

## Discussion

### Aspartic acid racemization

Aspartic acid racemization (AAR) is an analytical approach that uses the stereochemical conversion of aspartic acid to estimate the biological age of tissues. It is based on systematic sample preparation procedures, including amino acid extraction, protein hydrolysis, and derivatization for instrumental analysis. This approach is supported by chromatographic techniques, such as gas chromatography (GC) and high-performance liquid chromatography (HPLC), which separate and quantify amino acid enantiomers. Each technique has distinct applications, advantages, and limitations. Aspartic acid racemization has the primary benefit of using samples collected from tissue (dental) that have been shielded from numerous environmental and nutritional variables, giving a margin of error of approximately 1.5-4 years. ^10– 12^ The majority of organisms only have the L-form (levo) of optically active amino acids initially, which is then partially converted to the D-form (dextro) until equilibrium is attained over time or with aging, resulting in a mixture of the two forms called a racemic mixture.

The D/L ratio of aspartic acid is 1.0 in this equilibrium. This spontaneous conversion process is called racemization and causes a conformational change of a metabolically stable protein, thus driving a change in its activity and biochemical properties. This conversion stops at death (except when the body is placed in a hot environment), as the racemization rate relies on temperature, pH, and humidity, both in vivo and postmortem. ^10, 12– 16^

Racemization occurs in long-lived proteins. Aspartic acid has the fastest racemization rate, followed by alanine, glutamic acid, isoleucine, and leucine. Thus, age estimation methods use aspartic acid most often. ^10, 16, 17^ Modifications due to the instability of aspartate and asparaginyl residues in proteins may increase D-aspartate residues with age. Amino acids continuously undergo formation and degradation. In such cases, proteins with high metabolic rates provide less accurate results than tissues with low metabolic rates and long-lived proteins. ^10^

In humans, long-lived proteins with low metabolic rates can be found in the hard tissues of teeth, bones (type I collagen, osteocalcin, telopeptide), eye sclera (elastin), arterial walls (elastin), lung parenchyma (elastin), intervertebral discs, articular cartilage (proteoglycan), brain (tubulin, proteoglycan, synapsin, myelin basic protein, β-amyloid protein, white matter, tar protein), cartilage, ocular lens (αA-crystallin), erythrocyte membrane protein, epiglottis, and skin. Hard tissues, such as teeth and bones, remain well preserved after all soft tissues have deteriorated. Teeth are often well preserved even when large portions of bone have been mutilated or destroyed. ^10, 12^ The use of non-dental tissues such as cartilage, elastin, or bone in the aspartic acid racemization.

Several studies have analyzed various dental tissues such as enamel, dentin, cementum, or using the teeth. Dentin is the best tissue for age estimation and these results have been demonstrated in many populations. ^18^ Age and the degree of aspartic acid racemization in root dentin are strongly correlated (r=0.97 ± 0.01). Root dentin is considered to provide fairly accurate results because it is considered less affected by disease (e.g., caries), repair processes (e.g., presence of reparative dentin), or restoration. Dentin also has a greater proportion than enamel and is subject to fewer changes, such as attrition. The presence of caries on teeth will affect the level of racemization as caries will induce protein degradation, causing faster accumulation of D-aspartic acid. Therefore, the degree of racemization will be very high in samples taken from carious teeth, even if the sample comes from an area that is not affected by caries. Thus, it is not recommended to use carious tooth samples. Tooth samples that have been deposited for a long time may have smaller peptide fragments, leading to a more rapid conversion of the L-form to the D-form resulting in the higher accumulation of D-Asp. Ultimately, such protein degradation can produce high false-age estimates of death. ^3, 10, 12, 14, 19, 20^

Generally, aspartic acid racemization is quite complex, involving extracting the dentin from the tooth, powdering the dentin, and extracting the amino acids. Therefore, samples are analyzed by gas chromatography (GC), which is considered the most sensitive method. ^21^ The teeth’s position and the time required for dentine formation also affect the racemization rate. High-power liquid chromatography (HPLC) and gas chromatography-mass spectrometry (GC-MS) are widely used for analysis. ^10^ HPLC is considered an easier method for separating aspartic acid when compared to the GC method. Research conducted using the HPLC method shows that it can be effectively used to calculate chronological age by aspartic acid racemization. It can also deliver reliable results and only requires a short analysis time. ^5, 14^ However, the GC method is considered the most sensitive method. GC allows determining the quantitative ratio between the two isomers of the D-form and the L-form, which is then used in calculations. ^10, 13^

Several studies have compared the two techniques of GC and HPLC and observed the resulting advantages, stating that the HPLC technique is better for age estimation. However, other studies have provided better results through the GC technique. Therefore, neither of these techniques (HPLC or GC) is likely to be strictly more favorable than the other. ^18^ Expressing the racemization ratio as ln [(1 + D/L)/(1–D/L)] and placing this logarithmic racemization ratio on the y-axis and chronological age on the x-axis, a linear regression equation is obtained as

$$\mathrm{Ln}\;{\left[\left(1+D/L\right)/\left(1-D/L\right)\right]}_{t}=2\mathrm{kt}+\ln\;{\left[\left(1+D/L\right)/\left(1-D/L\right)\right]}_{t=0}$$

where *t* is the chronological age and *k* is the racemization rate constant. The subscripts *t = 0* and *t* for the logarithmic racemization ratio refer respectively to the status at the initial stage and at time *t.* The following equation can then be used to estimate chronological age ^10, 12– 16^:

$$t=\{\ln\;{\left[\left(1+D/L\right)/\left(1-D/L\right)\right]}_{t}–\ln\;{\left[\left(1+D/L\right)/\left(1-D/L\right)\right]}_{t=0}\}/2k.$$

Differences in environment, climate, genetics, race, nutrition, type of teeth used, part of the teeth used, temperature, humidity, and pH can cause different levels of racemization in each population. Temperature also has a huge effect on the D/L ratio. Moreover, the rate of aspartic acid racemization in tissues will increase with high temperatures; teeth that are located deeper in the oral cavity will also have a higher rate of racemization, because of the higher surrounding temperature. Previous studies have also shown that the older the sample, the greater the error. Age determination will be more accurate if it is done on samples younger than 35 years. ^3, 14^

It is also advisable to choose the incisors or premolars because both teeth are small, single-rooted, and easy to obtain enough dentin (overall) compared to molars with multiple roots. In tooth development, dentin is formed over years from the crown to the root apex concentric cones. In young people, the dentin D/L ratio is predicted to be larger in the crown than in the root. However, in elderly people, the opposite is true. ^11, 14^ However, there are also studies using third molars, arguing that these teeth are the last teeth to mineralize and grow, indicating that they were obtained from samples that were at least 18 years old. Moreover, they are less prone to caries and their position in the mouth protects them from environmental factors, making them ideal for research samples. ^16^

When compared with the DNA methylation method, aspartic acid racemization can still be used for archaeological sample analyses because the protein can be preserved well over a lengthy period while extracting sufficient intact DNA for DNA methylation analysis is very difficult. ^19, 22^

Aspartic acid racemization using bone samples is strongly influenced by the sex of the sample. Men’s bones are less affected by bone disease than women’s bones. Women’s bones are more affected by bone disease after menopause as the lack of estrogen leads to bone resorption and increased osteoclast activity. Consequently, typically the D/L ratio in female bones does not correlate with chronological age, especially after menopause. ^3^

The aspartic acid racemization method’s sensitivity to temperature is one of its drawbacks. Thus, it cannot be used for burnt samples or bodies that have been exposed to high temperatures. In addition, this is an invasive method that cannot be applied to individuals who are still alive. This method also often requires expensive instruments. ^12, 17^ Moreover, this method damages the sample, so care is needed when analyzing the samples that are available in limited quantities. ^23^ Age-related conditions brought on by protein aggregation, such as Alzheimer’s disease, cataracts, and arteriosclerosis, have also been linked to aspartic acid racemization reactions. ^21^

### DNA methylation

The process of altering the number 5’ carbon atom in a cytosine residue followed by a guanine residue is known as DNA methylation. Thus, it is known as a CpG(s)/CpG sites dinucleotide. Only at position 5 of the CpG dinucleotide sequence, in the pyrimidine ring of cytosine, is the replication process in DNA methylation found. The most accurate epigenetic alteration technique for determining the age of human biological samples is DNA methylation. In the genome, methylation (addition of a methyl group) occurs in a CpG-rich region (at the cytosine nucleotides in the cytosine/guanine region) called the CpG island. CpG islands are located on gene regulators such as promoters, intergenic regions, and repeat elements. Methyl-CpG domains and protein binding will bind specifically to the CpG island, and induce transcriptional activation and repression. ^7, 24, 25^ The level of total DNA methylation will decrease with increasing age of the individual. ^26^ DNA methylation is essential for several biological processes, including cell differentiation, embryonic development, and the regulation of gene expression. ^27^

The correlation between aging and DNA methylation can be explained in two ways: epigenetic drift and epigenetic clock. While the epigenetic clock is only weakly related to age, epigenetic drift reveals a variety of alterations in individuals related to age due to environmental influences. Therefore, it can be used to predict chronological age. In males and females, the CpG sites have different levels. Methylation on the X chromosome also tends to be more unstable. Women’s susceptibility to certain diseases and stress also greatly influences DNA methylation levels. Thus, many factors affect DNA methylation. Additionally, the metabolic syndromes and degenerative diseases of everyone also influence the percentage of DNA methylation. ^7^

There are three ways to detect and analyze DNA methylation: utilizing restriction endonuclease digestive enzymes, converting sodium bisulfite, and increasing affinity with methyl-binding proteins or CpG-specific antibodies. Bisulfite conversion represents a foundational chemical process in epigenetic analysis, enabling the distinction between methylated and unmethylated cytosines through selective deamination and thereby providing a reliable basis for downstream methylation profiling. The conversion of sodium bisulfite is a common procedure used in the field of forensics for the analysis and detection of DNA methylation. Examples of sodium bisulfite conversion procedures have been used in forensic odontology on teeth, saliva, and buccal swabs. Meanwhile, forensic examinations are generally conducted through blood, sperm, and urine. ^23^ The bisulfite conversion technique currently appears to be the most promising, although it requires a larger number of DNA samples than conventional forensic examinations. ^28^

Several markers related to age estimation have been researched for different tissues, including blood, buccal swabs, saliva, sperm, bone, and teeth, enabling these tissues to be used as highly accurate age prediction models (APM). ^29^ Subsequent amplification and detection employ techniques such as pyrosequencing, Sanger sequencing, EpiTyper, massive parallel sequencing or SNaPshot, and methylation-sensitive high-resolution melting (MS-HRM), each operating on distinct analytical principles to quantify methylation patterns with varying degrees of resolution and sensitivity. Collectively, these techniques offer critical implications for the accuracy and robustness of DNA-based forensic age estimation. Many methodologies are used for sequencing bisulfite targets including pyrosequencing, Sanger sequencing, EpiTyper, massive parallel sequencing or SNaPshot, and methylation-sensitive high-resolution melting (MS-HRM). ^27, 30^

Investigations most commonly use pyrosequencing. The primary advantage of pyrosequencing is the ability to obtain highly accurate quantitative DNA methylation data by the direct sequencing of PCR products. Nevertheless, there are limitations to this method, such as the relatively high cost and pyrosequencer’s low penetration rate. ^27^ Methylation levels can be quickly and relatively cheaply quantified using the PCR-based approach, MS-HRM. This technique compares the melt profiles of unknown PCR products with PCR products obtained from standards with known methylation ratios to determine DNA methylation levels. ^27^

Furthermore, CpG in the *FHL2* and *ELOVL2* genes has been linked to age estimation in numerous studies along with the markers *NPTX2*, *SCGN*, and *KLF14*. *ELOVL2* is a stable epigenetic marker. This locus was used as a marker that correlated strongly with age in many APMs obtained from specific tissues such as blood, teeth, bone, and buccal swabs, providing results with similar high accuracy across all APMs. A transcription co-factor called *FHL2* is involved in processes such as cell cycle regulation, wound healing, and bone formation. Gene expression is regulated in adipose tissue by the *KLF14* transcription factor. Other studies also mention that several other genes can also be used as markers in age estimation, among them are *EDARADD*, *PDE4C*, *C1orf132*, *TRIM59*, *PENK*, *TOM1L1*, *IASPA*, *TGA2B*, *CCDC102B*, *ZBF423*, *NHLRC1*, *SCGN*, *CSNK1D*, and *MIR29B2C*. ELOVL2 and TRIM59 gave better results as multi-tissue markers than *FHL2*, *KLF14*, and *C1orf132*. ^24, 28– 31^ The other most promising candidate gene is NRCAM, which displayed the strongest age correlation in both the validation and screening datasets (R ^2^ = 0.46 and R ^2^ = 0.84, respectively). ^28^

The right gene must be chosen to create a model for age estimate that is accurate. As *LOVL2*’s methylation rate has a strong, positive correlation with age, it has the highest potential for modeling age estimation. Other genes that are positively correlated are *PDE4C* and *FHL2*. Meanwhile, *EDARADD*, *ASPA*, *CCDC102B*, *C1orf132*, and *chr16:85395429* methylation rates are negatively correlated with age. ^27, 28^ Using DNA methylation levels for age estimation will give better results in samples with a younger age range. Conversely, there will be a reduction in accuracy (increase in MAD value) with age. ^29^

Several studies have shown a greater difference between chronological age and estimated age when using all tooth tissue for analysis compared to separating individual layers of the tooth. Differences in composition between layers and differences in cell types can affect the methylation status of selected genes. ^24^ A factor that can affect DNA methylation and must be considered before performing DNA analysis is the presence of some clinical conditions, diseases, or lifestyle habits such as drinking alcohol or smoking, which can interfere with methylation data. ^29^

Blood is utilized for DNA methylation-based age estimates since it is reasonably easy to collect. Teeth are more reliable sources of DNA than blood samples, even in badly decayed bodies (e.g., burnt cadavers, and white skeletons). Of the various types of teeth, molars provide the best protection for DNA. DNA is used for age estimation by analyzing its methylation level. ^27^ DNA in teeth originates from dentin (odontoblasts), dental pulp (immune cells, odontoblasts, fibroblasts, and undifferentiated mesenchymal cells), and cementum (cementocytes). In a study on 21 modern teeth from 7 to 77 years old, analysing methylation levels of *ELOVL2*, *FHL2*, and *PENK* genes, the median difference between chronological age and estimated age was smaller in pulp (2.25 y, SD = 2.5 y) and cementum (2.45 y, SD = 3.3 y) than in dentin (7.07, SD = 7.0). ^27^ Another study of 20 third molars from 22 to 70 years old subjects analysed CpG methylation levels of *ELOVL2*, *KLF14*, *SCGN*, *NTPX2*, and *FHL2* genes from pulp samples, using bisulfite-modified PCR and pyrosequencing. ^24^ Although the methylation status of individual genes and CpG sites provided at best relatively modest (though significant) correlation with age, multivariate models of combined genes with multiple CpG sites improved the correlations considerably and provided age estimates with mean absolute error (MAE) values of only 1.5-2.1 years. ^24^

The DNA content in premolars and molars is higher than in incisors and canines, this is possible because of the higher cellularity potential results from a relatively larger cementum size and larger pulp volume, more stable alveolar, and more anatomical protection. Thus, molars are more resistant to postmortem loss or contamination. Odontoblasts, as the principal cells in the pulp-dentin complex, degrade rapidly and disappear as soon as 5 days after death. Both cementum and pulp are major sources of dental genomic DNA. ^32^

DNA methylation analysis is now being used to estimate age for forensic identification, taking the place of earlier approaches. This procedure is very straightforward because it makes use of a readily available methylation kit. Additionally, the results can be obtained relatively fast and with a small number of samples. When compared to other DNA analysis methods like telomere shortening and sjTREC, DNA methylation examinations to estimate a person’s age also have the best correlation with chronological age. Currently, research on DNA methylation is still mostly performed with blood samples, but it has also begun to be conducted by taking samples from oral tissues, such as saliva, gingiva with swabs, and teeth. ^4, 23, 26, 31, 33^

## Conclusion

The method of analyzing the racemization of aspartic acid to estimate age has a high degree of accuracy, particularly in determining age at death. However, a limitation of this analysis method is that aspartic acid racemization is influenced by temperature and the condition of the teeth being sampled, such as caries. Given that DNA methylation is generally stable throughout a wide temperature range, it is possible to use this technique to determine the chronological age of even charred remains with satisfactory accuracy.

## Authors’ contributions

E.P., E.I.A, R.S.P., and A.G.A.P. conducted to study design and contributed to the initial draft of the manuscript. E.I.A., P. A, and A.W.S. supervised and approved the study. E.P. conducted the literature research and interpretation of data on the two compared methods, with R.S.P. and A.G.AP. as supporting observers. E.I.A., P. A, and A.W.S. contributed by critically revising the manuscript and approving the version for publication. All authors have read and approved the final manuscript.

## Ethics approval and consent to participate

Ethical approval and consent were not required.

## Consent for publication

Not applicable.

## Author details

^1^Forensic Odontology Specialist Study Program, Department of Oral Biology, Faculty of Dentistry, University of Indonesia, Indonesia.

^2^Division of Forensic Odontology, Department of Oral Biology, Faculty of Dentistry, University of Indonesia, Jakarta, Indonesia

^3^Master Programme in Dental Sciences, Division of Forensic Odontology, Faculty of Dentistry, University of Indonesia, Indonesia

^4^Master’s Programme In Biomedical Sciences, Faculty of Medicine, University of Indonesia, Jakarta, Indonesia

^5^Department of Mechanical Engineering, School of Engineering, Aalto University, Finland

## Data Availability

No data are associated with this article. Zenodo: Performance of Forensic Age Estimation by Aspartic Acid Racemization and DNA Methylation: A Systematic Review, DOI: https://doi.org/10.5281/zenodo.13624062. ^34^ The project contains the following reporting guidelines:
• Prisma Flowchart.jpg •
Checklist for Performance of forensic age estimation by aspartic acid racemization and DNA methylation a systematic review.docx Data are available under the terms of the Creative Commons Attribution 4.0 International license (CC-BY 4.0). Zenodo: Performance of Forensic Age Estimation by Aspartic Acid Racemization and DNA Methylation: A Systematic Review, DOI: https://doi.org/10.5281/zenodo.13624062. ^34^ The project contains the following extended data:
•

Table 1. Electronic literature search strategy.docx
•

Table 2. Journal synthesized results.docx Data are available under the terms of the Creative Commons Attribution 4.0 International license (CC-BY 4.0).
