# Supplementary material for: Performance of Forensic Age Estimation by Aspartic Acid Racemization and DNA Methylation: A Systematic Review
Source: F1000Res. 2026 May 14;13:1368. Originally published 2024 Nov 15. [Version 3] doi: 10.12688/f1000research.147348.3 (PMC13191321; doi:10.12688/f1000research.147348.3)
Supplement: Peer review history

# Reviewer report: Reviewer response for version 3

**DOI:** 10.5256/f1000research.198036.r485581

**Published:** 2026-05-20

**Authors:** Samiya Riaz

**Affiliations:** 1 International Islamic University Malaysia, Kuantan, Malaysia

The review is generally well-structured and follows PRISMA guidelines appropriately The inclusion of more recent studies (if available) beyond 2022 may further strengthen the comprehensiveness of the review. The discussion on environmental effects, particularly temperature influence on aspartic acid racemization, is informative. Adding a brief practical implication section, maybe one or two sentences,  for disaster victim identification scenarios would further enhance the manuscript. The discussion may benefit from inclusion of recent review articles focusing on modern forensic odontology techniques, imaging technologies, and AI-assisted identification approaches. The conclusion section is appropriate. Overall the manuscript is well written and is acceptable after minor revisions, which can then be reviewed by the editor only..

Are the rationale for, and objectives of, the Systematic Review clearly stated?

Yes

Is the statistical analysis and its interpretation appropriate?

Yes

If this is a Living Systematic Review, is the ‘living’ method appropriate and is the search schedule clearly defined and justified? (‘Living Systematic Review’ or a variation of this term should be included in the title.)

Yes

Are sufficient details of the methods and analysis provided to allow replication by others?

Yes

Are the conclusions drawn adequately supported by the results presented in the review?

Yes

Reviewer Expertise:

forensic odontology, human identification, dental profiling, tooth morphology

I confirm that I have read this submission and believe that I have an appropriate level of expertise to confirm that it is of an acceptable scientific standard.

## References

[1] : Advances in forensic odontology for human identification: a comprehensive review of methods, accuracy, and challenges. *IIUM Journal of Orofacial and Health Sciences* .2026;7(1) : 10.31436/ijohs.v7i1.409 70-80 10.31436/ijohs.v7i1.409

[2] : Modernizing Forensic Anthropology: A Data-driven Pipeline for Human Identification and Profiling. *European Journal of Dentistry* .2026;20(02) : 10.1055/s-0046-1816537 392-398 10.1055/s-0046-1816537 PMC1316060141740950

# Reviewer report: Reviewer response for version 1

**DOI:** 10.5256/f1000research.161535.r357357

**Published:** 2025-02-12

**Authors:** Fan Liu

**Affiliations:** 1 Naif Arab University of Security Sciences, Riyadh, Saudi Arabia

In this paper, the authors provide a systematic review comparing the performance of aspartic acid racemization (AAR) and DNA methylation methods in forensic age estimation. It adheres to the PRISMA guidelines and includes a robust search and selection process for relevant studies from 2017–2022. The manuscript offers insights into the accuracy, limitations, and practical applications of both methods, with data synthesized from 12 journal articles. The manuscript addresses a critical need in forensic science for reliable age estimation methods, especially in challenging scenarios such as charred or decomposed remains. The comparison of biochemical (AAR) and molecular (DNA methylation) approaches is timely and relevant. The inclusion of a clear PICO framework strengthens the systematic review process, and the use of PRISMA guidelines enhances transparency and reproducibility. The review evaluates mechanisms, advantages, and limitations of both methods, highlighting the stability of DNA methylation under extreme conditions and the high accuracy of AAR in controlled scenarios.

 The manuscript holds potential to make a significant contribution to forensic science. However, I have several concerns with the manuscript that I describe below.

 1. While the content is rich, the manuscript could benefit from improved organization. Results and discussions are often intermingled, reducing clarity. Tables summarizing key findings (e.g., comparison of methods, accuracy metrics, and study characteristics) are referenced but not integrated into the main text, making it difficult for readers to access essential information.

 2. The synthesis of findings from the 12 articles lacks critical evaluation. A detailed comparison of performance metrics, such as mean absolute error (MAE) and sensitivity to environmental factors, would strengthen the analysis. The discussion on population and sample source diversity, including geographic and demographic differences, is underexplored and would provide valuable context.

 3. While the authors mention a risk of bias assessment, specific findings or scores are not presented. Including a summary table of bias across the reviewed studies would improve transparency and credibility.

 4. The manuscript discusses limitations of the methods but provides limited guidance for forensic professionals on choosing between AAR and DNA methylation in specific scenarios. Practical recommendations based on findings would enhance the paper's utility.

 5. Throughout the manuscript, gene names (e.g., *ELOVL2*, *FHL2*, *NRCAM*) are not italicized. Gene names should consistently follow the standard scientific formatting.

 6. Some statistics are presented as intervals (e.g., 0.96–0.98) without clarifying whether these are ranges or two possible values. For consistency and clarity, statistics should be uniformly reported, with ranges clearly identified or converted into the mean ± SD format where applicable. Example sentences include:

- **Age and the degree of aspartic acid racemization in root dentin are strongly correlated (0.96–0.98)**.
- **multivariate models of combined genes with multiple CpG sites improved the correlations considerably and provided age estimates with mean absolute error (MAE) values of only 1.5–2.1 years**.

7. The regression equation for aspartic acid racemization presented in the manuscript,

 ln *1+D/L* *1-D/L* *t* *=2*kt *+*ln *1+D/L* *1-D/L* *t=0*e *,*

 contains an unexplained term (e). It is unclear whether (e) represents residual error or a typographical error. If the equation represents a fitted regression model, residuals should not appear. Furthermore, if the sentence containing the equation is not complete, a comma “,” should be added at the end of the equation. The authors should clarify this and consider following the equation format provided in Alkass et al. (2010), where this aspect is well-defined.

 8. In the DNA methylation section, the last sentence of the sixth paragraph states: “ **The other most promising candidate gene is NRCAM, which displayed the strongest age correlation in both the validation and screening datasets (R2 = 0.84 and R2 = 0.46, respectively).**” The order of the coefficients of determination should be corrected to (R2 = 0.46 and R2 = 0.84, respectively).

Are the rationale for, and objectives of, the Systematic Review clearly stated?

Yes

Is the statistical analysis and its interpretation appropriate?

Partly

If this is a Living Systematic Review, is the ‘living’ method appropriate and is the search schedule clearly defined and justified? (‘Living Systematic Review’ or a variation of this term should be included in the title.)

Partly

Are sufficient details of the methods and analysis provided to allow replication by others?

Partly

Are the conclusions drawn adequately supported by the results presented in the review?

Yes

Reviewer Expertise:

Bioinformatics; Biostatistics; Genomics; Epigenomics; Population genetics; Forensic DNA Phenotyping

I confirm that I have read this submission and believe that I have an appropriate level of expertise to confirm that it is of an acceptable scientific standard, however I have significant reservations, as outlined above.

## References

[1] : Age estimation in forensic sciences: application of combined aspartic acid racemization and radiocarbon analysis. *Mol Cell Proteomics* .2010;9(5) : 10.1074/mcp.M900525-MCP200 1022-30 10.1074/mcp.M900525-MCP200 19965905 PMC2871409

# Response

**Published:** 2025-02-18

**Authors:** Elza Ibrahim Auerkari

**Affiliations:** Oral Biology, University of Indonesia, Depok, West Java, Indonesia

We thank the reviewer for their insightful comments and constructive criticism, which will undoubtedly improve the quality and clarity of our manuscript. We address each point below:

1. Manuscript Organization:

We acknowledge that the organization of the manuscript could be improved. We will revise the manuscript to:

- Separate Results and Discussion: We will clearly separate the presentation of the results from their interpretation and discussion. This will involve restructuring the manuscript to have distinct "Results" and "Discussion" sections for each method (AAR and DNA methylation).
- Integrate Tables into Main Text: We will integrate the tables summarizing key findings into the main text. Instead of simply referencing them, we will refer to these tables specifically, highlighting the information they contain and discussing their implications. For example, we will state: "As shown in Table 1, the accuracy of AAR varies depending on the tissue type, with tooth dentin demonstrating the highest accuracy.
- Improved Flow: The manuscript will be read again for improved flow in general

 

1. Synthesis of Findings and Critical Evaluation:

We agree that a more critical evaluation of the synthesized findings is necessary. We will enhance the analysis by:

- Detailed Comparison of Performance Metrics: We will include a more detailed comparison of performance metrics (e.g., MAE, correlation coefficients) across the studies, explicitly addressing their sensitivity to environmental factors and other variables. This comparison will be presented in a revised and expanded table.
- Expanded Discussion on Population and Sample Source Diversity: We will expand the discussion on population and sample source diversity, including a more in-depth analysis of the geographic and demographic differences and their potential impact on the results.

 

1. Risk of Bias Assessment:

We acknowledge the need for greater transparency regarding the risk of bias assessment. We will include a summary table presenting the risk of bias scores for each reviewed study. This table will detail the specific domains assessed (e.g., selection bias, performance bias, detection bias) and the overall risk of bias judgment for each study.

  

1. Practical Guidance for Forensic Professionals:

We recognize the value of providing practical guidance for forensic professionals. We will expand the discussion to offer recommendations on choosing between AAR and DNA methylation based on specific scenarios. This will include considering factors such as:

- Sample Type and Condition: The type and condition of the available sample (e.g., charred, decomposed, dental tissue, bone).
- Environmental Factors: The potential impact of environmental factors (e.g., temperature, humidity) on each method.
- Required Accuracy Level: The desired level of accuracy for the age estimation.
- Available Resources: The laboratory equipment and expertise required for each method.

 

1. Gene Name Formatting:

We thank the reviewer for pointing out the inconsistencies in gene name formatting. We will carefully review the manuscript and ensure that all gene names (e.g., ELOVL2, FHL2, NRCAM) are consistently italicized throughout the text.

  

1. Statistical Reporting:

We appreciate the feedback on statistical reporting. We will revise the manuscript to:

- Clarify Intervals: Clearly identify whether the intervals presented are ranges or two possible values.
- Uniform Reporting: Ensure that statistics are uniformly reported, with ranges clearly identified or converted to mean ± SD format where applicable. For example, "Age and the degree of aspartic acid racemization in root dentin are strongly correlated (r = 0.97 ± 0.01, range 0.96-0.98)."

 

1. Regression Equation for Aspartic Acid Racemization:

We thank the reviewer for identifying the error in the regression equation. The term "e" was indeed a typographical error and will be removed. The corrected equation is:

 ln(1+D/L / 1-D/L)t = 2kt + ln(1+D/L / 1-D/L)t=0

 We will also clarify that the equation represents a fitted regression model and provide a more detailed explanation of the parameters, referencing the format used in Alkass et al. (2010) for consistency.

  

1. Order of Coefficients of Determination for NRCAM:

We acknowledge the error in the order of the coefficients of determination for NRCAM. We will correct the sentence to state: "The other most promising candidate gene is NRCAM, which displayed the strongest age correlation in both the validation and screening datasets (R2 = 0.46 and R2 = 0.84, respectively)."

 By addressing these points, we are confident that the revised manuscript will be a more valuable contribution to the field of forensic science. We appreciate the reviewer's time and effort in providing this valuable feedback.

# Reviewer report: Reviewer response for version 1

**DOI:** 10.5256/f1000research.161535.r353368

**Published:** 2025-01-25

**Authors:** Bofeng Zhu

**Affiliations:** 1 Xi'an Jiaotong University, Southern Medical University, Guangzhou, China

The author has meticulously conducted a bibliometric analysis of the application of aspartic acid racemization and DNA methylation in forensic age estimation, thereby providing a comprehensive overview of the current research landscape in this domain.

 Through this analysis, the author has systematically elucidated the principles, advantages, and limitations associated with the use of aspartic acid racemization and DNA methylation for age estimation in forensic science.

 The conclusions drawn from this study offer significant insights and valuable references for future research endeavors in individual age estimation. The study is undoubtedly of considerable significance.

 However, several suggestions are proposed to further enhance the rigor and clarity of the manuscript, which may be beneficial for its publication. Firstly, it is recommended that the bibliometric results be visually represented. Such visualization would facilitate a more immediate and accessible understanding of the data for readers.

 Secondly, the author should provide a detailed explanation of the criteria for inclusion and exclusion, including the specific references and rationale upon which these standards are based.

 This would enhance the transparency and reproducibility of the study. Lastly, a thorough discussion of the technological platforms underlying aspartic acid racemization and DNA methylation is warranted.

Are the rationale for, and objectives of, the Systematic Review clearly stated?

Yes

Is the statistical analysis and its interpretation appropriate?

Yes

If this is a Living Systematic Review, is the ‘living’ method appropriate and is the search schedule clearly defined and justified? (‘Living Systematic Review’ or a variation of this term should be included in the title.)

Partly

Are sufficient details of the methods and analysis provided to allow replication by others?

Yes

Are the conclusions drawn adequately supported by the results presented in the review?

Yes

Reviewer Expertise:

population genetics; forensic science; forensic genetics;

I confirm that I have read this submission and believe that I have an appropriate level of expertise to confirm that it is of an acceptable scientific standard, however I have significant reservations, as outlined above.

# Response

**Published:** 2025-02-18

**Authors:** Elza Ibrahim Auerkari

**Affiliations:** Oral Biology, University of Indonesia, Depok, West Java, Indonesia

We thank the reviewer for their thorough and constructive feedback, which we believe will significantly enhance the rigor and clarity of our manuscript. We address each point below:

1. Visual Representation of Bibliometric Results:

We agree that a visual representation of the bibliometric results would greatly improve the accessibility and understanding of our analysis. We will incorporate figures depicting:

- Publication Trends Over Time: A line graph illustrating the number of publications per year focusing on aspartic acid racemization and DNA methylation in forensic age estimation between 2017 and 2022.
- Keyword Co-occurrence Network: A network diagram displaying the relationships between keywords used in the reviewed articles, highlighting the most frequently studied aspects and emerging trends.
- Geographical Distribution: A map indicating the geographical distribution of research efforts in this field, showing the countries with the highest number of publications.

 

1. Detailed Explanation of Inclusion and Exclusion Criteria:

We acknowledge the need for a more detailed explanation of our inclusion and exclusion criteria to enhance the transparency and reproducibility of our study. We will revise the manuscript to include the following:

- Specific References: We will cite established guidelines and literature on systematic review methodology that informed our choice of inclusion and exclusion criteria.
- Rationale: We will provide a clear rationale for each criterion. For instance:

- Excluding "case reports, narrative reviews, controlled clinical trials, or case series": Because we intended to systematically synthesize original research findings on the accuracy and reliability of the two methods, rather than overview existing knowledge.
- Excluding "texts not available in full": Because to assess articles required a full text review, hence the exclusion.

- Clarity: We will ensure that the wording of the criteria is unambiguous and leaves no room for subjective interpretation.

 

1. Discussion of Technological Platforms Underlying AAR and DNA Methylation:

We appreciate the suggestion to provide a more thorough discussion of the technological platforms used for aspartic acid racemization and DNA methylation. In the revised manuscript, we will include sections detailing:

- Aspartic Acid Racemization:

- Sample Preparation Techniques: A description of the sample preparation steps involved in AAR, including the processes for extracting amino acids from tissues, hydrolyzing proteins, and derivatizing amino acids for analysis.
- Analytical Methods: Explanation on Gas Chromatography (GC) and High-Performance Liquid Chromatography (HPLC), which are commonly employed for the analysis of amino acid enantiomers, with examples of their applications, advantages, and limitations.

- DNA Methylation:

- Bisulfite Conversion: An explanation of the chemical process involved in bisulfite conversion, highlighting its significance in differentiating between methylated and unmethylated cytosines.
- Amplification and Detection Methods: Description on Pyrosequencing, Sanger Sequencing, MS-HRM and other commonly used methods for amplifying and detecting methylation patterns in DNA samples, including the principles behind each technique and their implications for forensic age estimation.

By incorporating these suggestions, we believe that the revised manuscript will be more comprehensive, transparent, and accessible to a wider audience, further enhancing its contribution to the field of forensic science.
